# Spontaneous epiretinal membrane resolution: mechanisms, outcomes, and implications for clinical management

**DOI:** 10.1007/s00417-025-06815-8

**Published:** 2025-03-31

**Authors:** Alberto Quarta, Maria Ludovica Ruggeri, Ruggero Tartaro, Lisa Toto, Rodolfo Mastropasqua

**Affiliations:** 1 Department of Neurosciences, Imaging and Clinical Sciences, University “G. d’Annunzio” Chieti-Pescara, Chieti, Italy; 2 Department of Medicine and Science of Ageing, Ophthalmology Clinic, University “G. d’Annunzio” Chieti-Pescara, Chieti, Italy; 3 Department of Sciences, Gabriele d’Annunzio University, Ophthalmology Clinic, National Center of High Technology in Ophthalmology, Via Dei Vestini, 66100 Chieti, Italy

**Keywords:** Epiretinal Membrane, Spontaneous resolution, Macular peeling

## Abstract

**Abstract:**

**Purpose:**

To review the phenomenon of spontaneous epiretinal membrane (ERM) resolution focusing on the clinical settings, mechanisms of resolution, and implications for management.

**Methods:**

A comprehensive review of case reports and studies describing spontaneous ERM resolution was conducted. Data were extracted regarding ERM type, patient demographics, visual outcomes, and suspected mechanisms. Findings were analyzed to identify trends and to compare cases of spontaneous resolution across different ERM types.

**Results:**

Spontaneous ERM resolution was more frequently observed in younger patients or cases associated with posterior vitreous detachment (PVD). In idiopathic ERMs, spontaneous separation often involved PVD and extracellular matrix remodeling. Inflammatory ERMs demonstrated resolution due to reduced inflammation and traction, while secondary ERMs linked to vascular or traumatic events benefited from mechanisms such as photocoagulation-induced PVD or gliotic contraction. Visual outcomes varied, with improvements in best-corrected visual acuity (BCVA) and metamorphopsia in most cases, though persistent structural changes occasionally limited functional recovery.

**Conclusion:**

Spontaneous ERM resolution is a rare but clinically significant event influenced by vitreoretinal interface dynamics and patient-specific factors. Younger age, the presence of PVD, and underlying inflammatory or vascular conditions may contribute to natural resolution. Recognizing these cases allows for tailored management strategies supporting observation in selected patients while minimizing unnecessary surgical interventions. These insights may guide future research into therapeutic approaches that mimic natural resolution mechanisms.

## Abstract (highlights)

**Key messages:**

*****What is known***:**

- Often associated with incomplete posterior vitreous detachment (PVD), these membranes show age-related changes in vitreoretinal adhesion and extracellular matrix dynamics.
- Inflammatory conditions, trauma, and vascular-related ERMs occasionally exhibit spontaneous peeling due to reduced traction or changes in the vitreoretinal interface.

*****What is new***:**

- Cases of inflammatory ERMs show that reduced cytokine levels and healing responses can facilitate natural detachment, highlighting the potential for conservative management in mild cases.
- Treatments such as photocoagulation and steroid injections, which induce PVD or reduce macular traction, appear to mimic natural separation processes offering new therapeutic insights.

## Introduction

Epiretinal membrane (ERM) is a fibrocellular proliferation on the surface of the retina, predominantly seen in older adults and often associated with posterior vitreous detachment (PVD), retinal vascular diseases, or inflammation [1]. While ERM typically progresses slowly causing symptoms such as metamorphopsia and reduced visual acuity, it has conventionally been managed through surgical intervention, particularly when it leads to significant visual impairment [2]. However, in rare cases, ERM can undergo spontaneous resolution, characterized by the detachment or retraction of the membrane [3–6].

The phenomenon is still largely unpredictable and rare, with an estimated incidence of 1–5.4% in idiopathic cases and slightly higher rates among younger patients with inflammation-associated ERM [7]. Most current knowledge is based on isolated case reports or small observational studies, which provide insufficient data to determine reliable predictive factors, underlying pathways or establish guidelines for clinical decision-making.

Consequently, it is unclear how often spontaneous separation occurs in each disease context, what specific clinical or anatomical characteristics might predispose a patient to this phenomenon. This gap in the literature limits the potential for non-surgical management strategies as most clinicians currently lack the evidence needed to assess whether a patient might benefit from a period of observation rather than immediate surgery. Understanding the potential for spontaneous separation could help refine clinical decision-making.

Given these knowledge gaps, a comprehensive review of cases to map out the phenomenon could significantly contribute to disease management. In this context, understanding spontaneous separation is not only clinically relevant but could also reduce surgical risks, healthcare costs, and patient burden.

## Methods of search

We conducted a literature search using the PubMed, Medline, and Embase databases, covering the period from January 1990 to October 2024. The review period starts from 1990 due to the critical transition from film-based to digital Color Fundus Photography (CFP) and the following introduction of OCT into clinical practice, which enhanced objective assessment. The search terms utilized encompassed various relevant terms such as ‘’Spontaneous ERM resolution’’, “Spontaneous ERM peeling’’, ‘’Spontaneous ERM separation’’, ‘’Spontaneous ERM release’’, ‘’Spontaneous macular pucker resolution’’, “Spontaneous macular pucker peeling’’, ‘’Spontaneous macular pucker separation’’, ‘’Spontaneous macular pucker release’’. All articles identified using the specified keywords were thoroughly reviewed by two reviewers (A.Q., L.T.). Any disagreements were resolved through discussions between the two reviewers, with the involvement of a third reviewer (R.M.) when necessary. Articles not written in English were excluded, and duplicate entries were removed. Abstracts of unpublished studies and review studies were also not included. The inclusion criteria for the narrative review consisted of studies and cases that examined or described a spontaneous ERM resolution. Studies were excluded if they lacked detailed descriptions of the clinical case, lacked imaging description with fundus camera or OCT, did not include patient-level data, or if the title or abstract did not provide sufficient information. In such cases, the full text was reviewed and screened for the inclusion criteria. This review encompassed 36 articles which were confirmed after excluding duplicates, further excluding articles based on title, abstract, or language criteria.

### Data collection

The following data were collected: authors' names and year of publication, setting, patient age, follow-up to ERM resolution, initial and final BCVA, final metamorphopsia and suspected mechanism of resolution. All these parameters were reported in Table 1. If any of these parameters were not identified in the examined articles, it was recorded in the tables as “not specified”. BCVA from each article was converted in logMAR for clarity. We divided the cases according to the setting.

**Table 1:** Clinical scenarios reported for ERMs

Author (Year)	Setting	Patient Age (years)	Follow-up to ERM Resolution	Initial BCVA (logMAR)	Final BCVA (logMAR)	Final Metamorphopsia	Suspected Mechanism of Resolution
Idiopathic ERM							
Meyer CH et al. (2004)	Idiopathic ERM	15–30 years	2–13 months	0.2–0.8	Improved	Resolved	Stronger contractile forces in immature membranes overpowered retinal adhesions; dynamic remodeling of extracellular matrix facilitated separation
Gao H et al. (2007)	Idiopathic ERM (pediatric)	7 years	3 months	0.5	0.1	Improved	Immature vitreoretinal interface facilitated PVD-induced separation; dynamic remodeling led to resolution
Cakir M et al. (2007)	Idiopathic ERM (Stargardt)	15 years	4 months	1.0	0.7	Improved	Cellular remodeling and tangential traction resolved ERM associated with Stargardt dystrophy
Kolomeyer AM, Schwartz DM (2013)	Idiopathic ERM	68 years	12 months	0.5	0.3	Improved	PVD facilitated ERM peeling by reducing vitreoretinal traction; conservative observation allowed natural progression
Mansour AM et al. (2014)	Idiopathic ERM	32 years	2 months post-weight lifting	Visual loss	0	Resolved	Valsalva maneuver-induced intraocular pressure increase caused central ERM rupture, facilitating spontaneous separation
Pierru A et al. (2016) (Case 1)	Idiopathic ERM	35 years	Not specified	0	0	Improved	Spontaneous separation facilitated by glial contraction or PVD
Pierru A et al. (2016) (Case 2)	Idiopathic ERM and POAG	58 years	Not specified	0	0	Improved	Mechanical stress by a bympy tractor ride triggered PVD, leading to ERM release
Menteş J, Nalçacı S (2020)	Idiopathic ERM	56 years	1 week	0	0	Resolved	Anteroposterior traction from vitreous detachment exceeded adhesion forces, leading to ERM separation as a floating flap
Xue-Qing B et al. (2021)	Idiopathic ERM (pediatric)	3 years	2 months (recurrence resolved in 3 months)	Improved	Improved	Improved	Immature ILM defects allowed glial proliferation and contraction; spontaneous separation linked to dynamic remodeling and PVD formation
Kamada R, Iwase T (2021)	Idiopathic ERM with macular pseudohole	66 years	2 months	0	−0.12	Resolved	ERM contraction and PVD-induced pulling forces caused separation
Gupta R et al. (2023) (Case 1)	Idiopathic ERM	66 years	Gradual over multiple visits	Stable floaters	Stable floaters	Not applicable	Dissolution of ERM matrix over time, likely due to extracellular matrix remodeling
Gupta R et al. (2023) (Case 2)	Idiopathic ERM	62 years	51 months	Stable floaters	Stable floaters	Not applicable	Gradual regression and thinning of the ERM matrix without clear detachment
Gupta R et al. (2023) (Case 3)	Idiopathic ERM with macular hole	66 years	3 months	Blurred vision	Improved	Improved	PVD-induced traction led to vitreomacular release and ERM separation
Zeng QZ, Yu WZ (2023)	Idiopathic ERM	33 years	2 months (recurrence observed at 3 months)	0	0	Resolved	Initial PVD and tangential traction caused separation; recurrence linked to residual membrane on ILM or new glial proliferation
Inflammatory ERM							
Schadlu R et al. (2007)	Inflammation-associated ERM	16 years	5 months	0.5	0.1	Resolved	Pre-existing PVD facilitated ERM separation; reduction in inflammatory activity allowed natural regression
Gupta A et al. (2012)	Inflammatory ERM (toxoplasmosis)	24 years	~ 3 weeks	0.3	0	Resolved	Younger age with dynamic ERM cellularity; spontaneous PVD induced ERM contraction and separation during healing
Guemes-Villahoz N et al. (2023)	Inflammatory ERM (uveitis cases)	9–53 years	2–12 months	Variable	Near-normal	Improved	Cellular changes in inflammation-driven ERM; PVD facilitated separation; inflammatory cells led to weaker attachment
Retinal tumor associated ERM							
Manjandavida FP et al. (2014)	Secondary to VPT	Median 44 years	~ 5 years (mean follow-up)	Range 0–1.0	Improved (31%)	Improved or stable	Cryotherapy-induced inflammation and gliosis caused ERM contraction and release
Kolomeyer AM et al. (2016)	Secondary to VHL hemangiomas	23 years	After 4 sessions of FPAL	0.1	0.1	Resolved	Fluorescein-potentiated argon laser (FPAL) induced PVD and disrupted vitreoretinal interface, facilitating peeling
Kumar V (2017)	Secondary to hamartoma	Not specified	Not specified	Not specified	Not specified	Not specified	Hamartoma-associated contraction of ERM
Ozgonul et al. (2018)	ERM in peripheral hemangioma	60	Not specified	0.1	0	Not specified	Tangential cell-mediated traction due to reduction in exudates/swelling; increased contractility led to membrane separation from retina
Ding X et al. (2022)	Secondary to retinal vascular tumor	Mean 33.6 years	Varied across 8 cases	Improved in 5 cases	Stable or improved in others	Not specified	Photocoagulation-induced PVD and increased glial contraction led to ERM peeling
Tong L et al. (2022)	Secondary to VPTR	56 years	~ 2 sessions of LP	1.0	1.0	Improved	Laser photocoagulation (LP)-induced PVD and retinal gliosis increased ERM contractility, leading to peeling
Munoz-Solano J et al. (2024)	Secondary to RH	25 years	1 month post-treatment	1.0	1.0	Resolved	PDT-induced inflammation and phototoxic effects caused PVD and localized ERM peeling
Ding X et al. (2022)	Secondary to retinal vascular tumor	Mean 33.6 years	Varied across 8 cases	Improved in 5 cases	Stable or improved in others	Not specified	Photocoagulation-induced PVD and increased glial contraction led to ERM peeling
Miscellaneous secondary ERM							
Sugimoto M et al. (2002)	Secondary to Coats’ disease	Not specified	Not specified	Variable	Improved	Improved	Photocoagulation around aneurysms triggered PVD and spontaneous ERM peeling
Murata T et al. (2007)	Secondary to Leber’s aneurysms	17 years	1 month post-treatment	0.2	1.0	Improved	Photocoagulation-induced partial PVD caused ERM peeling; mild vitreous inflammation may have contributed
Wetzel et al. (2016)	Optic nerve atrophy	56 years	2 years	0.7	1.0	Not specified	Optic nerve atrophy led to RNFL thinning, reducing glial contractility and weakening ERM adhesion
Pierru A et al. (2016) (Case 2)	Traumatic ERM	15 years	10 months post-trauma	0	0	Improved	Trauma-induced mechanical stress triggered PVD, leading to ERM release
Andreev et al. (2016)	Secondary to BRVO	65 years	Not specified	0.2	0	Not specified	Myofibroblast contraction and progressive separation without visible defects
Mizobuchi et al. (2022)	Secondary to Coat’s disease	10 years	4 months	0.2	0	Resolved	PVD-related detachment
Alshahrani et al. (2022)	Secondary to BRVO with macular edema	86 years	6 months	1.3	0.1	Improved	Steroid-induced reduction of macular edema altered tangential traction, facilitating ERM separation
Kanai et al. (2024)	Secondary to RRD repair	20 years	1 year	0	0	None	Contractile forces of ERM and post-laser photocoagulation effects. Boxing-related trauma may have contributed

### Cases description

Spontaneous regression of ERM has been reported across a wide spectrum of clinical scenarios. This section provides a detailed account of previously documented cases to highlight the diversity of presentations and hypothesized pathways leading to spontaneous ERM separation.

### Idiopathic ERM

Meyer et al. (2004) describe six young patients aged 15–30 years with idiopathic ERM. Spontaneous separation occurred in all patients over a follow-up period of 2–13 months [8]. ERM separation was attributed to the strong contractile forces of immature membranes in younger patients, potentially driven by cellular remodeling and dynamic extracellular matrix changes. A similar case during childhood was described by Gao et al., who reported the spontaneous separation within three months from initial observation [9]. In this case, the authors proposed that separation was favoured by the attachment of the ERM to the cortical vitreous, which detached as part of vitreous development in children.

Over four months of observation Cakir et al. reported a 15-year-old boy with Stargardt disease who showed a spontaneous ERM separation, leading to BCVA improvement [10]. The separation was attributed to cellular remodeling and tangential traction forces in the setting of retinal degeneration.

Beyond childhood, the spontaneous resolution was described in a 68-years old man over 12 months of observation, resulting in BCVA improvement [11]. The separation was likely facilitated by PVD-induced tractional forces, reducing adhesion between the ERM and retina.

Mansour et al. (2014) described the case of a 32-year-old weightlifting athlete who experienced a gradual visual improvement in the right eye two months after noticing visual loss. Examination revealed a partially separated ERM with a rolled-over edge, attributed to the effects of a Valsalva maneuver performed during weightlifting [12]. The increased intraocular pressure from the maneuver likely stressed the ERM, causing it to rupture at its weakest adhesion points. Similarly, Pierru et al. reported two cases emphasizing the role of PVD, trauma, or external forces in facilitating separation [13].

Time of resolution seems variable, and a spontaneous resolution within a week of follow-up to two months was also reported [14, 15]. The detachment was attributed to anteroposterior tractional forces exerted by the detached vitreous, which overcame ERM adhesion.

Spontaneous resolution was described in a 3-year-old girl within two-months after strabismus surgery, with recurrence at one year and resolution again within three months [16], demonstrating that after resolution the occurrence is already possible.

More extensive follow-up was described for spontaneous resolution, and description of a 62-year-old woman with constant floaters in the left eye was observed over 51 months, during which the ERM gradually regressed without detachment, resulting in anatomical normalization. Beyond time for follow-up, the phenomenon was described also with VMT and associated ERM [17].

The initial separation is likely facilitated by PVD, but as previously described in a childhood case, a recurrence in adult is possible, probably due to residual membrane remnants and glial cell proliferation on the ILM [18].

### Inflammation-induced ERM

Inflammation-induced ERM has been documented in younger patients, often resolving alongside the resolution of the inflammatory process.

Schadlu and Apte (2007) describe a 16-year-old male with intermediate uveitis and a pre-existing PVD, who developed an ERM in the left eye. Over five months, the ERM spontaneously resolved without surgical intervention [19]. The authors propose that the initial PVD combined with subsiding inflammation facilitated the ERM’s detachment, marking a rare instance of spontaneous resolution associated with pre-existing PVD.

A treatment-related resolution was described in a case of toxoplasmic retinochoroiditis. After treatment with antibiotics and corticosteroids, the ERM spontaneously separated as inflammation subsided. OCT confirmed complete separation of the ERM along with the development of PVD, and visual acuity improved [20]. The authors attribute the resolution to the patient’s younger age, a more dynamic cellular environment, and reduced inflammation, which allowed the ERM to contract and detach from the retina.

Similarly to idiopathic ERM cases, spontaneous resolution was described within 2 to 12 months of follow-up for a Bechet uveitis. The authors hypothesize that the higher proportion of inflammatory cells in these ERMs compared to idiopathic ERMs may weaken retinal adhesion and promote spontaneous separation [21]. These findings suggest that conservative management might be appropriate for inflammatory ERMs, particularly in younger patients or those with mild visual impairment. Given these characteristics, inflammation-driven ERMs may regress once the primary inflammation resolves or when the vitreous undergoes structural changes, as seen in cases with induced or spontaneous PVD. This cellular and biochemical flexibility may explain why younger patients with active inflammation have higher rates of spontaneous ERM regression compared to older patients with idiopathic ERM.

### ERM with Retinal Tumor

ERM in the context of retinal vasoproliferative tumors (VPTs) and retinal capillary hemangiomas shows a unique clinical challenge. The pathogenesis in these cases is likely multifactorial, involving chronic low-grade inflammation, exudation, and glial cell proliferation, which collectively contribute to a pro-fibrotic state.

Manjandavida et al. (2014) analyzed 16 eyes with retinal VPT and associated ERMs treated with cryotherapy. In 63% of cases, spontaneous ERM separation occurred post-treatment. Cryotherapy-induced inflammation and tissue contraction were thought to enhance gliosis, leading to ERM release [22]. While most patients showed stable or improved vision, a small subset experienced worsened outcomes due to complications.

The treatment may influence spontaneous resolution. In von Hippel-Lindau (VHL) disease, bilateral retinal hemangiomas after fluorescein-potentiated argon laser therapy (FPAL) to treat the hemangiomas, the ERM separated spontaneously [23]. The proposed mechanism involves FPAL-induced PVD and possible laser-induced contraction of retinal tissue, which weakened ERM adhesions.

A similar case without treatment was reported in combined hamartoma of the retina and retinal pigment epithelium [24]. The mechanism suggested includes the contractile forces of the ERM surpassing the adhesion forces, potentially aided by dynamic vitreoretinal changes.

In a case of retinal hemangioma with spontaneous ERM separation over three years, the resolution was linked to reduced traction forces due to the regression of retinal exudates and macular edema (ME), as well as the influence of PVD [25].

Tong et al. (2022) describe a 56-year-old male with a retinal VPT and secondary ERM in the left eye. The patient underwent two rounds of laser photocoagulation. Following the second round, OCT revealed spontaneous ERM separation, coinciding with VPT regression and the development of PVD [26]. This case suggests that laser-induced PVD and increased contractility in the ERM may facilitate spontaneous resolution. This mechanism is further investigated in a series of eight cases, suggesting that laser-induced heat and inflammation lead to PVD, as well as enhanced glial and retinal pigment epithelial cell activation which promotes ERM contraction and peeling [27].

Munoz-Solano et al. (2024) detail a 25-year-old male with retinal hemangioblastoma and secondary ERM. The patient received anti-VEGF therapy followed by photodynamic therapy (PDT), resulting in spontaneous ERM separation [28]. PDT likely triggered localized inflammation and PVD, facilitating the peeling of the ERM. Anti-VEGF therapy may have further supported this process by modifying the vitreoretinal interface.

### Miscellaneous conditions associated with secondary ERM

Miscellaneous conditions related to vascular diseases, post-surgical clinical scenarios and degenerative diseases may be the setting for a spontaneous ERM resolution.

The case described by Sugimoto (2002) involves a 26-year-old male with untreated Coats' disease who presented with visual deterioration and ERM [29]. The author described the spontaneous separation of ERM after laser photocoagulation, possibly due to induced PVD.

Murata et al. (2007) present the case of a 17-year-old male with Leber’s multiple miliary aneurysms who developed a secondary ERM in the right eye. Argon laser photocoagulation was performed to treat capillary nonperfusion around the aneurysms. Within a month, the ERM spontaneously separated, leaving a gliotic mass and restoring visual acuity [30]. The authors suggest that partial PVD induced by photocoagulation created tractional forces that led to ERM peeling.

Andreev et al. (2016) report a case of a 65-year-old woman with secondary ERM following branch retinal vein occlusion (BRVO). Initially, she exhibited incomplete PVD and peripheral retinal neovascularization. Opting for observation rather than immediate surgery, the ERM gradually separated over time [31]. The release was attributed to myofibroblast contraction, potentially aided by mechanical forces and gradual PVD development.

Mizobuchi and colleagues (2022) described a 10-year-old Japanese boy with stage 2 Coats disease, characterized by ERM, retinal telangiectasia, microaneurysms, hard exudates, and peripheral retinal hemorrhages. Over two months, OCT revealed spontaneous detachment of the preretinal macular fibrosis, with complete peeling observed at four months [32]. PVD expanded and appeared to facilitate the detachment of the thick posterior vitreous membrane associated with fibrosis. No exudative or tractional detachment occurred over a long-term follow-up of nearly five years. The rapid reduction in macular swelling can relieve the tangential traction exerted by the ERM, leading to avulsion or spontaneous peeling of the membrane.

Wetzel et al. (2016) describe a 56-year-old male with a history of alcohol abuse and optic nerve atrophy who experienced a gradual, spontaneous regression of idiopathic ERM over two years. The ERM caused retinal distortion and mild thickening in the right eye, but as the optic nerve atrophied and retinal nerve fiber layer thinned, the ERM regressed, nearly restoring normal macular thickness. However, the patient’s visual acuity decreased from 20/100 to 20/200 due to ongoing optic nerve damage [33]. The regression mechanism is attributed to reduced glial cell activity and diminished contractile forces as a result of optic nerve atrophy.

Alshahrani et al. (2022) report an 86-year-old diabetic male with BRVO who declined surgical treatment for a grade 2 ERM associated with ME. Instead, the patient received a single dexamethasone implant injection, which reduced ME and led to partial ERM separation after six months [34]. The mechanism behind this spontaneous separation involved the steroid’s anti-inflammatory effects, which reduced tangential tractional forces through the rapid resolution of edema.

In a 20-year-old male boxer case, the patient developed a secondary ERM after undergoing pars plana vitrectomy (PPV) for rhegmatogenous retinal detachment (RRD) caused by ora serrata dialysis. Over the course of a year, OCT revealed progressive ERM release without intervention [35]. The separation likely resulted from tangential tractional forces, possibly influenced by postoperative contractile forces, laser photocoagulation for securing the tear, and trauma associated with the patient’s boxing activities.

ERM regression was already described also in the context of specific systemic therapies. Lai et al. describe a 44-year-old female patient with idiopathic ERM spontaneous regression under retinoic acid therapy [36]. They suggested that retinoic acid likely promoted inhibition of the cellular and extracellular components that contribute to ERM contractility and adhesion. Confalonieri et al. report a 58-year-old woman with progressive ERM resolution during a 2-years follow-up, without BCVA changes [37]. The mechanism responsible for ERM separation was attributed to Angiotensin receptor blockers (ARBs) for arterial hypertension and migraine.

## Discussion

Spontaneous ERM regression is an intriguing phenomenon that has been observed across a range of demographic groups and clinical contexts. Each subset provides insights into possible mechanisms especially regarding why ERMs spontaneously resolve in some cases while persisting in others.

**Fig. 1:**
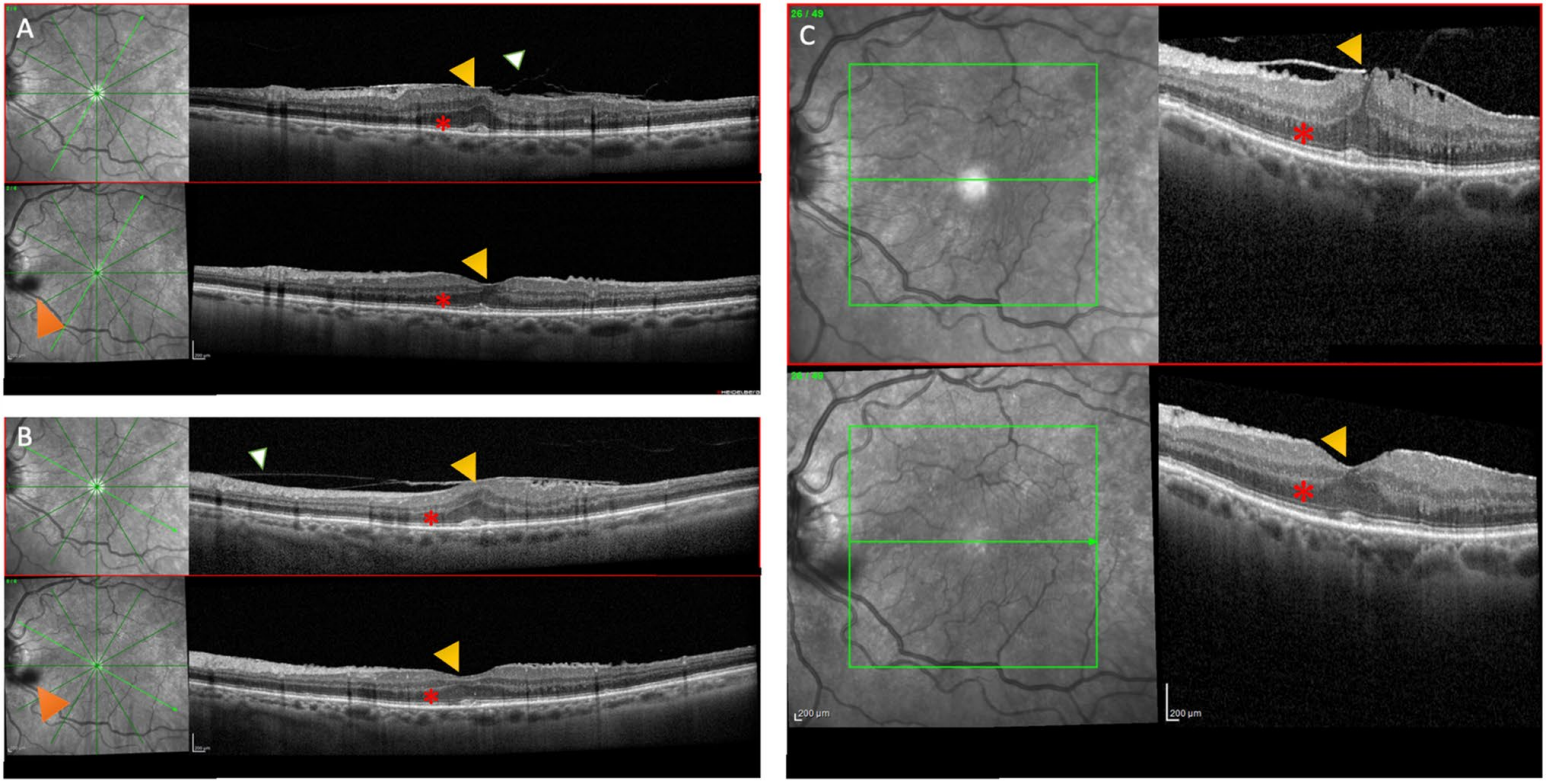
Optical coherence tomography at baseline and 6-months follow up of a spontaneous ERM resolution case. In radial scans comparison (**A**), it can be noted stage 3 ERM and vitreous adhesion (*white triangle*). Also, Ectopic Inner Foveal Layer (EIFL) and central foveal bouquet abnormality can be appreciated. In comparison (**B**), a different radial scan highlights posterior vitreous cortex splitting (*white triangle*) with membrane attachment. Comparison (**C**) shows dense scans highlighting regression of the foveal distortion. In all scans are reported EIFL (*yellow triangle*) and central bouquet abnormality regression (*red asterisk*) from baseline to 6-months visit. Orange triangle shows a vitreal shadow probably reflecting a fluctuating peeled ERM

Spontaneous resolution of idiopathic ERMs often involve dynamic structural changes at the vitreoretinal interface such as partial PVD [15, 17] (Fig. 1). Younger patients demonstrate a greater tendency for ERM resolution due to a potentially less robust cellular attachment and a different biochemical profile at the vitreoretinal interface [8–16]**.** Follow-up ranged from 2 months to over 4 years**,** with variable BCVA improvement and resolution of metamorphopsia. Suspected mechanisms included stronger contractile forces in younger patients leading to self-peel, extracellular matrix remodeling, and mechanical stress (e.g., Valsalva maneuvers or trauma) [12, 13]. Attention should be paid to those patients experiencing significant and worsening visual impairment accompanied by metamorphopsia and the potential development of amblyopia; in these cases, PPV combined with membrane peeling should be considered [38]. However, in older patients, age-related changes in cell adhesion and vitreous dynamics seems to reduce the likelihood of spontaneous separation [11, 18]. Future prospective studies could focus on identifying biochemical markers that predict ERM resolution in younger patients.

Inflammatory stimuli contribute to ERM development, but spontaneous regression is rare and occurs mainly when inflammation is well controlled [39]. Follow-ups ranged from weeks to 12 months, with reported BCVA improvement and resolution of metamorphopsia. These cases often involve pre-existing PVD or reductions in inflammatory activity, suggesting a strong interplay between inflammation, cellular remodeling, and mechanical forces in driving separation [19, 20]. However, the risk of recurrence necessitates long-term follow-up. Further research is needed to explore the role of anti-inflammatory therapies in modulating ERM behavior and improving visual outcomes.

ERMs related to retinal tumors have been observed to regress following treatments such as photocoagulation or cryotherapy. Reducing the tumor's vascular contribution and associated exudation may alter tractional forces or biochemical signaling that support ERM adherence [22, 28]. Reported follow-ups extend up to 5 years, with varying degrees of BCVA improvement. Younger patients show a greater tendency for ERM regression, suggesting a potential age-related factor. Mechanisms of resolution include tumor treatment-induced PVD, reduction in vascular exudation, and changes in extracellular matrix composition. The variability in ERM response to tumor-targeted treatments suggests that individual tumor characteristics, such as size, location, and vascular activity, influence the likelihood of spontaneous regression [23–27]. Future studies should investigate the specific tumor-related cytokines that influence ERM development and regression.

**Fig. 2:**
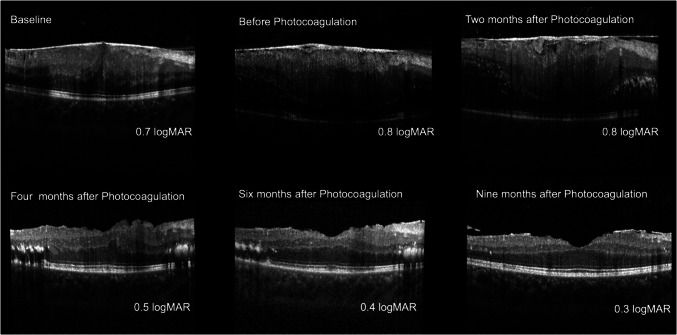
A case of ERM in Coats disease that spontaneously resolved following argon laser photocoagulation. Each panel represents sequential OCT scans, with corresponding logMAR visual acuity measurements. Baseline (Top Left)**:** The OCT scan shows significant macular thickening due to the presence of an ERM, with a visual acuity of 0.7 logMAR. Before Photocoagulation (Top Center)**:** Persistent ERM with unchanged macular thickening and worsen retinal layer segmentation, visual acuity worsened to 0.8 logMAR. Two Months After Photocoagulation (Top Right)**:** Slight reduction in membrane thickness, visual acuity remains 0.8 logMAR. Four Months After Photocoagulation (Bottom Left)**:** Substantial improvement in macular architecture with visible peeling of the ERM, visual acuity improved to 0.5 logMAR. Six Months After Photocoagulation (Bottom Center)**:** Further restoration of normal macular contour, with a visual acuity of 0.4 logMAR. Nine Months After Photocoagulation (Bottom Right)**:** Complete resolution of the ERM and restoration of macular anatomy, accompanied by significant improvement in visual acuity to 0.3 logMAR

ERMs associated with retinal vascular diseases and conditions such as Leber’s aneurysms or Coats’ disease often regress after therapeutic interventions including photocoagulation and steroid treatments. [29–32] (Fig. 2). In post-RD repair cases, laser photocoagulation and trauma-induced PVD may contribute to detachment [13, 30, 35]. Reported follow-ups vary widely, from 1 month to several years, with some cases showing BCVA stabilization or improvement. Pharmacological agents such as retinoic acid and ARBs have shown potential in reducing extracellular matrix growth and cellular contraction forces, facilitating ERM detachment [36, 37]. Prospective studies should evaluate the long-term efficacy of these pharmacological agents in promoting ERM regression.

Mechanisms and clinical outcomes of spontaneous ERM separation were recently investigated in small studies [7, 40]. Eyes with PVD showed more gradual ERM separation driven by tangential forces, while those without PVD had abrupt separations often associated with vertical traction. Post-separation visual improvement was limited (mean logMAR improvement of 0.08), with inner segment/outer segment (IS/OS) junction defects being a significant predictor of visual outcomes [7]. Lee et al., with a smaller cohort of 50 eyes, found a slightly higher incidence of ERM self-resolution, likely due to prolonged follow-up. Their study identified PVD as the main mechanism in 82% of cases. Interestingly, idiopathic ERMs resolved faster than secondary ones (23.4 vs. 34.1 months), but visual improvement was more pronounced in secondary ERMs, particularly in eyes with lower baseline visual acuity (odds ratio 267.6). As previously discussed, these works suggest that hypothesized mechanism of resolution may rely on PVD, tangential membrane contraction, inflammatory mediators and age-related factors.

The randomized controlled trial by Kofod et al. provides valuable insights into the timing of surgical intervention for ERMs in patients with good presenting visual acuity and mild symptoms [41]. The study compared immediate vitrectomy to a watchful waiting approach, with follow-ups extending up to three years for some patients. Results showed that visual acuity gains were similar between the immediate surgery group and the watchful waiting group after one year, suggesting that deferring surgery can be a safe and viable option for many patients. Choi et al. found in a retrospective study spotted choroidal changes in ERM spontaneous resolution, pointing at a new potential biomarker [42]. Kida et al. found different tryptase activity in the vitreous of spontaneous ERM separation compared to the vitreous of patients with proliferative diabetic retinopathy, hypothesizing a potential tryptase role in vitreous dynamics [43]. Despite these encouraging outcomes, functional recovery after ERM regression is not always guaranteed. The duration of the disease plays a significant role, as long-standing ERMs can cause irreversible photoreceptor damage limiting the potential for visual recovery even after successful peeling. Additionally, residual traction or incomplete separation of the membrane can lead to persistent retinal distortion or prevent full restoration of visual acuity. Coexisting pathologies such as ME, retinal vascular abnormalities, or optic nerve atrophy, may further restrict visual improvement despite the regression of the ERM.

Metamorphopsia is another critical parameter that often improves with spontaneous ERM separation. Nomoto et al. noted that reduced metamorphopsia scores post-resolution aligned with restored foveal contours on OCT, indicating that macular architectural integrity plays a significant role in alleviating distortion [44]. In inflammatory cases, distortion improvement depends on the resolution of traction and the absence of structural macular damage.

Observation without immediate intervention may provide the same results of immediate surgery. Moreover, follow-up is crucial in the eventual detection of visual symptoms worsening in the case of progression despite spontaneous resolution. However, the conclusions raised from this review only indicate the initial characteristics of cases that were spontaneously resolved. This does not mean that a specific case with these characteristics is more likely to spontaneously resolve. Moreover, the analysis does not reflect the natural course of cases that were severely symptomatic and required early surgery, and some of the discussed mechanisms remain hypothetical. This suggests that clinical and instrumental considerations should be made before deciding on surgery or observation.

Prospective studies are needed to understand the real need of surgical intervention.

## Data Availability

The data that support the findings of this study are available from the corresponding author upon reasonable request.
